# Aberrant salience network and its functional coupling with default and executive networks in minimal hepatic encephalopathy: a resting-state fMRI study

**DOI:** 10.1038/srep27092

**Published:** 2016-06-01

**Authors:** Hua-Jun Chen, Qiu-Feng Chen, Jun Liu, Hai-Bin Shi

**Affiliations:** 1 Department of Radiology, The First Affiliated Hospital of Nanjing Medical University, Nanjing 210029, China; 2 School of Information Science and Engineering, Central South University, Changsha 410083, China

## Abstract

The purposes of this study are to explore functional alterations in salience network (SN) and its functional coupling with default mode (DMN) and central executive (CEN) networks in minimal hepatic encephalopathy (MHE). Twenty cirrhotic patients with MHE, 23 cirrhotic patients without MHE (NHE), and 18 controls underwent resting-state fMRI and psychometric hepatic encephalopathy score (PHES) test. Independent component analysis was performed to obtain DMN (including three subsystems: anterior, inferior-posterior, and superior-posterior DMN [a/ip/spDMN]), SN, and CEN (including three subsystems: left-ventral, right-ventral, and dorsal CEN [lv/rv/dCEN]). The intrinsic functional connectivity (iFC) within (intra-iFC) and between (inter-iFC and time-lagged inter-iFC) networks was measured. MHE patients showed decreased intra-iFC within aDMN, SN, lvCEN, and rvCEN; and decreased inter-iFC and time-lagged inter-iFC between SN and ipDMN/spDMN/lvCEN and increased inter-iFC and time-lagged inter-iFC between SN and aDMN, compared with controls. A progressive trend in connectivity alterations was found as the disease developed from NHE to MHE. The inter-iFC between ipDMN/spDMN and SN was significantly correlated with PHES score. In conclusion, an aberrant SN and its functional interaction with the DMN/CEN are core features of MHE that are associated with disease progression and may play an important role in neurocognitive dysfunction in MHE.

Minimal hepatic encephalopathy (MHE) is a common neurocognitive complication of cirrhosis associated with increased progression into overt hepatic encephalopathy (HE) and a poor prognosis^1^,^2^, diminished quality of life^3^, and impaired driving skills, as well as a high motor-vehicle accident risk^4^,^5^. MHE results in a specific spectrum of high-level neurocognitive deficits, including attention deficits, working memory problems, and defects in executive functions such as response inhibition^6^,^7^,^8^,^9^. However, the mechanisms underlying these neural dysfunctions remain incompletely understood.

The science of large-scale brain networks has offered new ways of understanding human brain cognition procedures and investigating dysfunctional brain architecture in neuropsychological disorders^10^. Recently, there is increasing evidence that the systematic investigation of distinct large-scale neurocognitive networks can provide an important perspective to uncover mechanisms regarding high-level neurocognitive dysfunction^11^.

For example, a unifying “triple network” model proposed recently, including the salience network (SN), the default mode network (DMN), and the central executive network (CEN), is a representative paradigm. This model highlights the crucial role of the SN in initiating network switching that can lead to the engagement of the CEN and the disengagement of the DMN during the response to a salient stimulus. Recent studies have demonstrated that analysis of the triple network model is helpful in synthesizing disparate findings into a common framework and better explaining the mechanisms underlying neuropsychological disorders, such as Alzheimer’s disease^12^, Parkinson’s disease^13^, major depressive disorder^14^, and schizophrenia^15^.

The SN, composed of the bilateral anterior insular cortex (AI) and dorsal anterior cingulate cortex (ACC), is involved in the detection of and orientation to salient external stimuli and internal events^16^. The CEN consists mainly of the dorsolateral prefrontal cortex (DLPFC) and posterior parietal cortex (PPC), while the DMN includes primarily the medial prefrontal cortex (MPFC) and posterior cingulate cortex (PCC). Typically, the CEN represents a task-positive network, which is always activated in response to external stimuli; whereas the DMN consistently shows deactivation and is called as a “task-negative” network^17^,^18^. The SN (with the right AI as the integral causal outflow) facilitates switching activity between the DMN and CEN, through initiation of appropriate transient control signals that engage the CEN to mediate cognitive control processes while disengaging the DMN^16^. Currently, the preservation of functional interactions among the three networks is considered to be essential to focus attention on external stimuli and to complete various cognitive functions smoothly^11^. Notably, the functional coupling and anti-correlated coupling among triple networks have been demonstrated to be critically involved in the brain’s attentional, working memory and executive control processes (11, 16), all of which have been found to be impaired in MHE^6^,^7^,^19^.

As described previously^20^,^21^,^22^, MHE is associated with disturbed intrinsic large-scale brain networks such as the DMN and CEN. However, few studies have examined alterations in SN function and its interactions with the distinct networks, although this may provide new insight into MHE-related mechanisms from a more comprehensive perspective. Both functional and structural changes in the AI (the pivotal area of the SN) have been demonstrated in a few previous studies in MHE. Chen *et al.*^23^ reported that cirrhotic patients can show reduced gray matter volume in the bilateral AI and dorsal ACC. Also, Qi *et al.*^24^ revealed decreased functional activity within the bilateral AI of MHE patients.

Given this context, we hypothesized that the functional organization of the SN and its functional interactions with the CEN and DMN are altered in MHE, which could be correlated with neurocognitive dysfunction and disease progression. Therefore, we examined functional connectivity (FC) within the SN and between the SN and CEN/DMN for the first time. Given the prominent role of the SN in modulating DMN/CEN activity^15^,^16^,^25^, we also assessed the temporal dependencies of the SN on the DMN/CEN networks. Finally, Pearson’s correlation analyses were performed to examine the relationship between these connectivity alterations and neurocognitive dysfunction assessed by psychometric hepatic encephalopathy score (PHES).

## Materials and Methods

### Participants

This study was approved by the Research Ethics Committee of The First Affiliated Hospital of Nanjing Medical University, and all experiments were performed in accordance with relevant guidelines and regulations. The written informed consent was obtained from all participants. Twenty cirrhotic patients with MHE, 23 cirrhotic patients without MHE (NHE), and 18 healthy controls (HCs) were included. Psychometric hepatic encephalopathy score examinations, including five subtests—digit symbol test, number connection test A, number connection test B, serial dotting test, and line tracing test, were used to identify MHE. Details of the PHES tests have been described previously^26^. MHE was diagnosed when PHES performance was impaired by two standard deviations beyond normal performance^27^,^28^,^29^. Thus, a diagnosis of MHE was made when the PHES score was ≤−5.0 points. Subjects with current overt HE or other neuropsychiatric disorders and those taking psychotropic medications, suffering from uncontrolled endocrine or metabolic diseases, or with a history of alcohol abuse during the 6 months prior to the study were excluded.

### MRI data acquisition

We acquired all MRI data using a 3.0 T scanner (Siemens, Verio, Germany). Resting-state functional images were acquired using an echo planar imaging sequence with the following parameters: 35 contiguous axial slices, TR = 2000 ms, TE = 25 ms, FOV = 240 mm × 240 mm, matrix = 64 × 64, flip angle = 90°, and slice thickness = 4 mm. Participants were instructed to keep their eyes closed, to not think of anything in particular, and to keep their heads still. In addition, three-dimensional T1-weighted magnetization-prepared rapid gradient echo (MPRAGE) sagittal images were collected using the following parameters: TR = 1.9 ms, TE = 2.48 ms, FOV = 256 mm × 256 mm, matrix = 256 × 256, flip angle = 9°, slice thickness = 1.0 mm, 176 slices.

### VBM Analysis

VBM analysis was performed using the Statistical Parametric Mapping (SPM8, http://www.fil.ion.ucl.ac.uk/spm) software and the VBM8 toolbox (http://dbm.neuro.uni-jena.de/vbm.html). The diffeomorphic anatomical registration using exponentiated lie algebra (DARTEL) registration method was applied. Briefly, after segmentation of structural T1 images into gray matter (GM), white matter, and cerebrospinal fluid, GM maps were normalized to the GM population-specific template generated from the complete image set using DARTEL^30^. Spatially normalized images were then modulated to preserve the overall amount of GM tissue. Here, we considered only non-linear volume changes, so that further analyses did not have to account for differences in head size. The GM scores were included as covariates of no interest in the functional analyses, as described previously^15^, given that both network-specific intra-network intrinsic functional connectivity (intra-iFC) and inter-network intrinsic functional connectivity (inter-iFC) are thought to depend on widespread integrity of polysynaptic pathways. Additionally, the functional images were normalized into Montreal Neurological Institute (MNI) space by applying transformation parameters obtained from the unified segmentation procedure for the structural images.

### Functional image preprocessing

Functional data were preprocessed using the SPM software and the Data Processing Assistant for Resting-State fMRI (DPARSF, http://www.restfmri.net/forum/DPARSF) tool. The first 10 volumes were discarded to allow for scanner calibration and the adaptation of participants to the scanning environment. The remaining volumes were then corrected for differences in slice acquisition times and were realigned to correct for head movements. Subjects with more than 3-mm maximum displacement in any of the *x*, *y*, or *z* directions or more than 3.0° angular rotation about any axis would have been excluded from the study, but no participant had to be excluded; there were no significant differences regarding translational or rotational movements in any direction across the three groups (*P* > 0.05). Finally, all of the corrected functional data were normalized to MNI space and resampled to a 3-mm isotropic resolution and smoothed using a Gaussian filter with full-width at half-maximum of 6 mm.

### Independent component analysis (ICA)

Group spatial ICA was performed using the Infomax algorithm within GIFT software (http://icatb.sourceforge.net). A two-step principal component analysis (PCA) was used to decompose the preprocessed data into 75 independent components (ICs). In this study, we applied the high-model-order ICA approach, following recent studies^15^,^31^. The high-model-order ICA approach is considered to yield ICs that are in accordance with known anatomical and functional segmentations and can offer a more detailed and robust decomposition of sub-networks^31^,^32^,^33^. We ran 20 ICA (ICASSO implemented in GIFT software) to ensure the stability of the estimated ICs. This resulted in a set of average group components, which were then back-reconstructed into single subject space.

To select networks of interest, we ran multiple spatial regressions on the spatial maps of 75 ICs using the previously established T-maps of intrinsic connectivity networks^15^,^31^, which were made available online by the Medical Image Analysis Lab (http://mialab.mrn.org/data/hcp/RSN_HC_unthresholded_tmaps.nii). Of these established T-maps, seven are related to the “triple network” model, including anterior DMN (aDMN), inferior-posterior DMN (ipDMN), superior-posterior DMN (spDMN), left-ventral CEN (lvCEN), right-ventral CEN (rvCEN), dorsal CEN (dCEN), and SN. These seven subsystems are in accordance with the known anatomical and functional segmentations of DMN/CEN/SN^31^,^32^,^33^ and have been consistently identified throughout the distinct studies and used to explore the mechanisms underlying various neuropsychological disorders, such as Alzheimer’s disease^12^, Parkinson’s disease^13^, major depressive disorder^14^, and schizophrenia^15^. Thereby, following previous studies, the seven ICs with the largest correlation coefficients were selected as the networks of interest for our triple-mode network analysis, i.e. aDMN, ipDMN, spDMN, lvCEN, rvCEN, dCEN, and SN.

### Intra-iFC

Each IC obtained by the ICA procedure consisted of a spatial Z-map reflecting the component’s functional connectivity pattern across space and an associated time course (TC) reflecting the component’s activity across time. The Z values indicate the fit of a specific area’s TC to the group-averaged component’s TC, representing functional connectivity strength within the network (intra-iFC).

The ICs corresponding to the triple networks were then extracted from all subjects. The Z-maps of each network were gathered in each group for a random-effect analysis using a one-sample t-test (*P* < 0.05, false discovery rate (FDR) correction). Subsequently, the Z-maps of each network were compared across three groups using one-way analysis of variance (ANOVA), with age, gender, education level, and GM volume as covariates of no interest. A statistical map was generated using an integrated threshold at *P* < 0.05 (corrected by Monte Carlo simulations^34^). The group comparisons were restricted (masked) to the voxels within each corresponding network.

### Inter-iFC

Before evaluating inter-iFC statistically, linear detrending and temporal filtering were performed on the functional data. Then, we extracted the subject IC’s TCs of the seven sub-networks. The pair-wise Pearson’s correlation coefficients of the TCs among the seven sub-networks were calculated and transformed to Z-scores using Fisher’s Z-transformation and compared across the three groups, with age, gender, education level, and GM volumes as covariates of no interests (*P* < 0.05, FDR correction).

### Time-lagged inter-iFC

It has been documented that the SN is an integral hub in mediating dynamic interactions between the CEN and DMN^11^. In this study, we hypothesized an altered temporal interaction between SN’s preceding the TC and DMN/CEN TCs in MHE. Thus, a time-lagged correlation analysis was performed to investigate temporal dependencies between the SN and CEN/DMN subsystems^15^, with a time shift of 1 to 3 time points (See supplemental material)^15^. The Pearson’s correlation coefficients obtained were then transformed to Z-scores using Fisher’s Z-transformation and compared across the three groups, with age, gender, education level, and GM volumes as covariates of no interest (*P* < 0.05, FDR correction).

### Correlation analysis

The altered intra-iFC, inter-iFC, and time-lagged inter-iFC were extracted and correlated to the PHES result using Pearson’s correlation analyses. *P* values < 0.05 were considered to indicate statistical significance.

## Results

### Demographic and clinical characteristics of the subjects

Table 1 shows the subjects’ demographic and clinical parameters. There was no significant difference in terms of age, gender, or education level across the three groups. The NHE patients showed a trend of impaired neurocognitive function, compared with HCs, whereas MHE patients produced significantly worse performances in all PHES subtests, resulting in lower PHES score, as compared to the other two groups.

### Intra-iFC comparison

**Figure 1:**
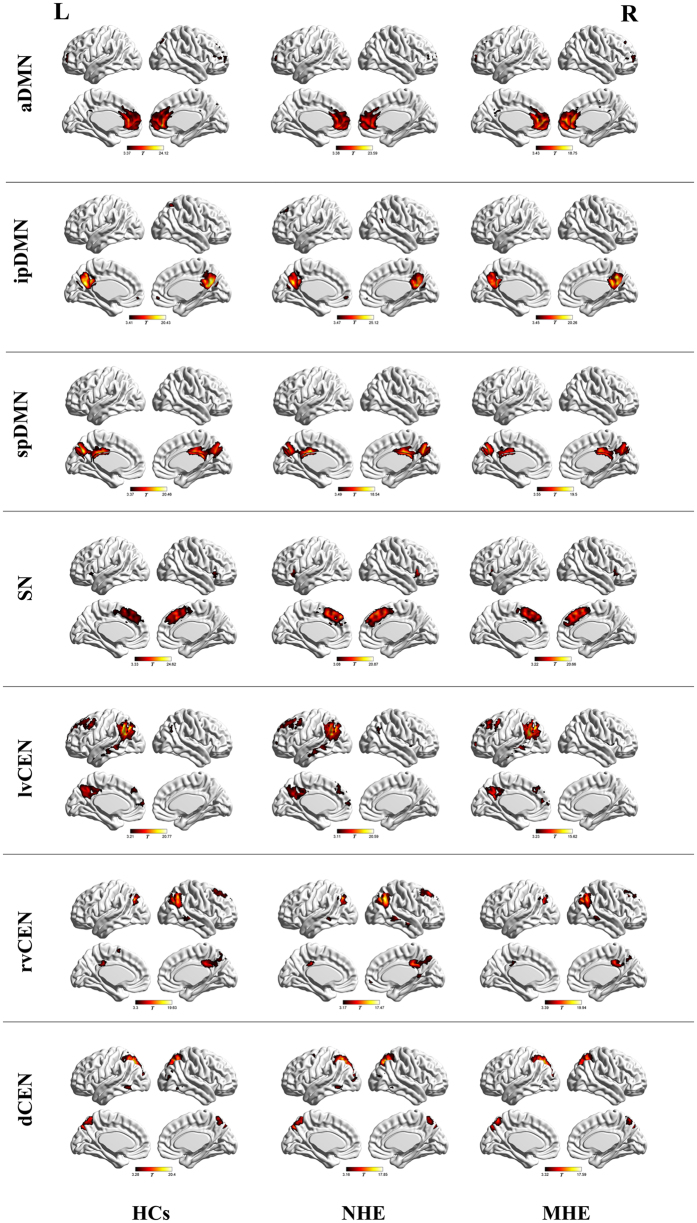
Spatial maps of the triple intrinsic networks. MHE, minimal hepatic encephalopathy, NHE, patients without MHE, HCs, healthy controls. a/ip/spDMN, anterior/inferior-posterior/superior-posterior DMN; lv/rv/dCEN, left-ventral/right-ventral/dorsal CEN; SN, salience network.

Random-effect analyses of single-subject IC maps revealed the typical spatial patterns of the SN, DMN, and CEN across the three groups (Fig. 1), which matched previously established templates and were consistent with other studies^14^,^15^,^31^. The SN was represented in one component, which showed functional connectivity between the dorsal ACC/MPFC and bilateral AI. Both the DMN and CEN were represented in three components. The aDMN included mainly the MPFC; the ipDMN consisted of the medial PPC, PCC, and angular gyrus; and the spDMN included primarily the bilateral precuneus and PCC. The lvCEN consisted mainly of the left inferior parietal lobule (IPL) and left DLPFC, while the rvCEN included primarily the right IPL and right DLPFC. The dCEN was left-lateralized, comprising primarily the left superior parietal gyrus/precuneus and right superior parietal gyrus/precuneus.

**Figure 2:**
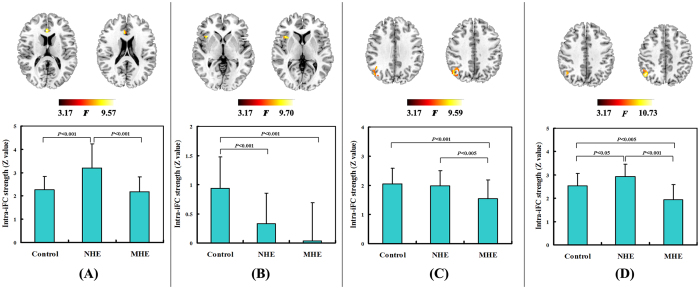
Significant differences in intra-network functional connectivity across the three groups. The brain areas with significantly altered intra-network functional connectivity are shown: (**A**) within aDMN, (**B**) within SN, (**C**) within lvCEN, and (**D**) within rvCEN. The Bar graphs show the results of *post hoc* comparisons between each pair of groups. Z values are derived from independent component analysis and indicate the functional connectivity strength within the network.

By comparing the mean intra-iFC values of the whole intrinsic network, we found that MHE patients showed decreased trends in intra-iFC for all DMN, SN, and CEN networks (see Supplementary Fig. 1 in supplemental material). Voxel-wise ANOVA revealed differences of intra-iFC within the aDMN (located in the bilateral ACC), SN (located in the right AI), lvCEN (located in the right IPL), and rvCEN (located in the right IPL) across the three groups (Fig. 2, Table 2). No between-group difference was observed within other networks. Both the MHE and NHE groups showed decreased intra-iFC in the SN and lvCEN regions, compared with HCs. Furthermore, the decrease in intra-iFC intensified as the disease progressed (from NHE to MHE), suggesting continued impairment of functional integration. Regarding the aDMN and rvCEN regions, MHE showed decreased trends for intra-iFC, while NHE showed increased intra-iFC.

### Inter-iFC comparison

**Figure 3:**
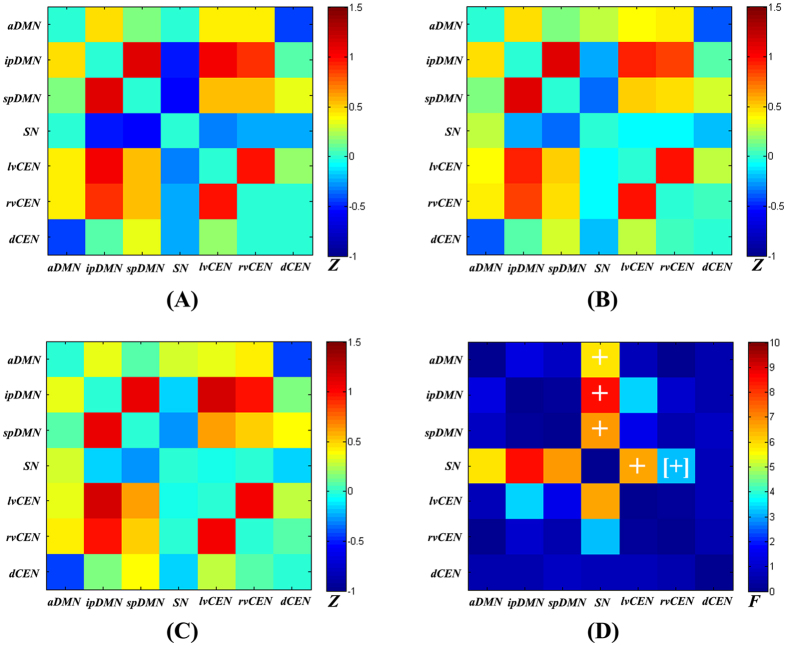
Inter-network intrinsic functional connectivity matrix. Pair-wise Pearson’s correlations between the time courses of the triple networks were Fisher Z-transformed and averaged across subjects for each group: (**A**) healthy controls, (**B**) NHE patients, and (**C**) MHE patients. The intensity of colors in the correlation matrix indicates the averaged Z-score. (**D**) The statistical results by one-way analysis of variance. The marker + indicates a significant difference across the three groups (*P* < 0.05, FDR correction). The marker [+] indicates a difference across the three groups (*P* < 0.05, uncorrected).

Figure 3a shows the inter-iFC matrix for HCs, which matched results of previous studies^14^,^15^,^31^,^35^. Positive correlations were found between distinct DMN/CEN subsystems, consistent with recent findings by a high-model-order ICA approach^14^,^15^,^31^,^35^, although this result contrasts with the patterns of anti-correlation between the two networks previously described in other studies^18^. In fact, it has been proposed that the DMN can be subdivided into several functionally distinct sub-networks, each with its own characteristic patterns of correlations and anti-correlations with other intrinsic networks^36^.

**Figure 4:**
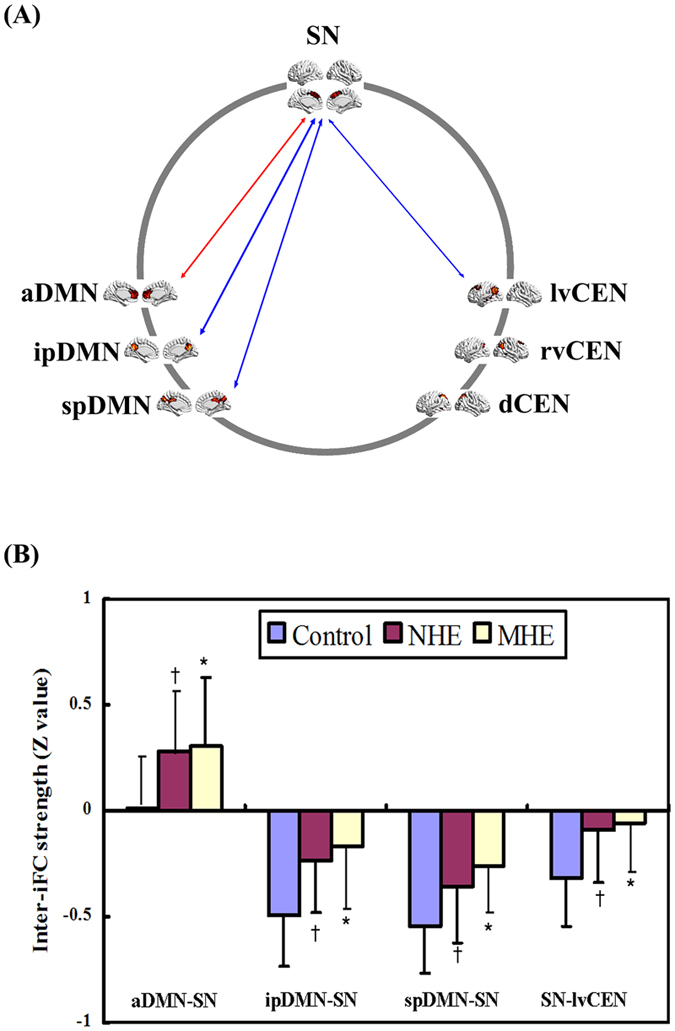
Between-group differences of inter-network functional connectivity. Spatial maps (derived from one-sample t-test for the control group) indicate the anterior/inferior-posterior/superior-posterior DMN (a/ip/spDMN), left-ventral/right-ventral/dorsal CEN (lv/rv/dCEN), and salience network (SN). (**A**) Red and blue two-way arrow indicate increased positive inter-network connectivity and decreased negative inter-network connectivity in MHE and NHE patients, respectively. (**B**) The markers * and ^†^indicate a significant difference of inter-iFC between MHE and HC and between NHE and HC, respectively.

Altered inter-network functional connectivity was found between the a/ip/spDMN and SN and between the SN and lvCEN (Fig. 3, Table 3). Figure 4 shows increased positive inter-network connectivity between the aDMN and SN in patients. Decreased negative inter-network connectivity was found between the ipDMN and SN, spDMN and SN, and SN and lvCEN. Furthermore, these alterations showed a trend with progression from NHE to MHE. Additionally, we found that the inter-iFC between ipDMN-lvCEN differed across the three groups, but this change did not reach statistical significance after FDR correction (Fig. 3, Table 3).

### Time-lagged inter-iFC comparison

**Figure 5:**
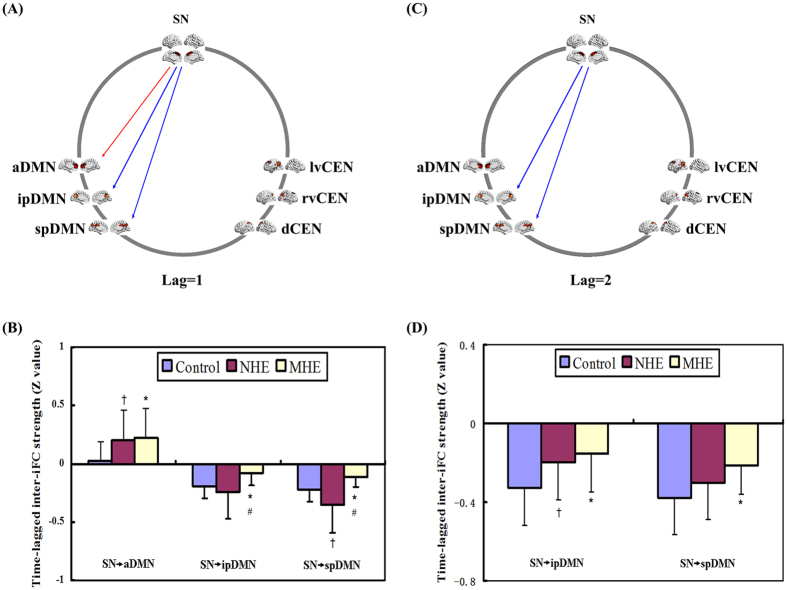
Between-group differences of time-lagged inter-network functional connectivity. Spatial maps (derived from one-sample *t*-tests for the control group) indicate the anterior/inferior-posterior/superior-posterior DMN (a/ip/spDMN), left-ventral/right- ventral/dorsal CEN (lv/rv/dCEN), and salience network (SN). (**A**,**C**): Red and blue one-way arrows indicate increased positive connectivity and decreased negative connectivity (centered on SN) in MHE patients, respectively. (**B**,**D**): The markers *, ^†^, and ^#^indicate significant differences between MHE and HCs, NHE and HCs, and NHE and MHE, respectively.

For lag = 1, the time-lagged inter-iFC of the SN onto the ipDMN/spDMN decreased in the MHE group, while the time-lagged inter-iFC of the SN onto the aDMN increased, compared with HCs (Fig. 5, Table 4). For lag = 2, the cirrhotic patients showed reduced time-lagged inter-iFC of the SN onto the ipDMN/spDMN, which progressed from NHE to MHE. For lag = 3, there was no significant difference in time-lagged inter-iFC across the three groups.

### Correlation analysis

**Figure 6:**
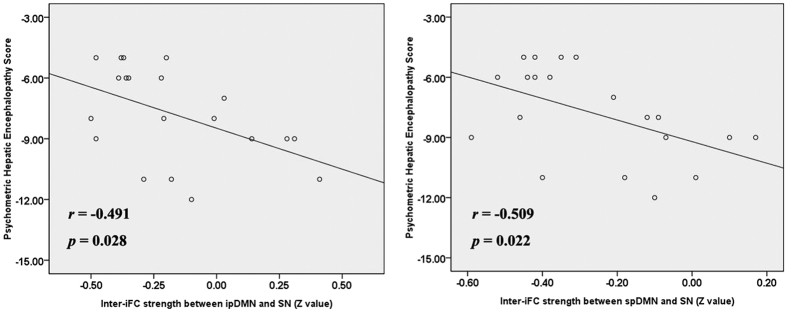
Scatter plots of PHES scores and inter-network functional connectivity in the MHE group. ip/spDMN, inferior-posterior/superior-posterior DMN; SN, salience network.

We first extracted the values of the intra-iFC (from the areas shown in Fig. 2), inter-iFC (shown in Fig. 4), and time-lagged inter-iFC (shown in Fig. 5) and then correlated them to PHES. Figure 6 shows the results of the correlation analysis between altered connectivity and impaired neurocognitive performance. In MHE group, the inter-network connectivities between the ipDMN/spDMN and SN were negatively correlated with PHES scores. No correlation was found between altered intra-iFC/ time-lagged inter-iFC and PHES result.

## Discussion

In this study, we investigated the FC alterations within and between the SN, CEN, and DMN in MHE patients, based on a prior theory of a triple-network mode^11^. The primary findings included: (i) MHE patients showed decreased intra-iFC in the aDMN, SN, lvCEN, and rvCEN; (ii) MHE patients showed an increased positive correlation between the aDMN and SN and decreased negative correlations between the ipDMN and SN, spDMN and SN, and SN and lvCEN; (iii) the time-lagged inter-iFC (centered on SN) was decreased between the ipDMN and SN and between the spDMN and SN, whereas it was increased between the aDMN and SN in MHE patients; (iv) the altered functional correlation between the ipDMN/spDMN and SN was correlated with impaired neurocognitive performance in MHE group; and (v) the progressive changes of intra-iFC and inter-iFC were found as the disease developed from NHE to MHE. Our results provide the first evidence about atypical connectivity patterns across the core neurocognitive networks in MHE, which may further elucidate the mechanisms that underlie MHE.

Impairments in several high-level neurocognitive domains comprise the characteristic manifestations of MHE. For example, attention deficit is one of the main components of MHE^19^. The anterior attention system, which is hypothesized to modulate response inhibition, behavioral selection, and executive control, is sensitive to early HE^37^. In fact, impaired executive function is well documented in MHE^6^,^9^. Correspondingly, recent resting state functional network analyses have revealed connectivity disruptions within the intrinsic brain networks related to those neurocognitive functions affected by MHE, such as the default-mode, attention, and executive networks^20^,^21^,^26^. Consistent with these previous findings, we also found altered intra-iFC in the aDMN, lvCEN, and rvCEN in MHE.

More importantly, we found altered SN and its functional coupling with the DMN and CEN. The SN-related controlling model, which engages the brain’s attentional, working memory and higher-order control processes while disengaging other systems that are not immediately task-relevant, has been proposed recently. This represents the optimal organization of cognitive resources and is essential for responding to salient stimuli and successfully completing various core neurocognitive functions^11^,^16^. Based on the triple network analysis, we found alterations in functional coupling between the SN and DMN/CEN in cirrhotic patients. The decreased coupling between the SN and CEN/DMN could indicate functional impairment in MHE, while the increased FC has been regarded as a compensatory reallocation or recruitment of cognitive resources, as previous reports in MHE^22^,^26^,^38^. Beyond the reports about single network analysis, our results can provide more comprehensive insight into the mechanisms about MHE, because triple network analysis can offer opportunity to organize the findings about altered networks into a systematical framework. In fact, analysis of complex brain networks has revealed that MHE is associated with aberrant connectivity patterns at the whole-brain scale, such as impaired small-world network efficiency and altered modular organization of intrinsic networks^39^,^40^,^41^, rather than with aberrant connectivity at a single-network level. Additionally, Chen *et al.*^21^ revealed that the investigation of multiple intrinsic brain networks is more helpful to depict the clinical state of MHE. In consideration of these previous studies, it is expected that changes of functional coupling among SN/CEN/DMN triple networks occur in MHE.

The NHE subjects, who represented an intermediate stage of disease, also showed an impaired trend in neurocognitive functions and intra- and inter-network FC across SN/DMN/CEN. Thus, we illustrated that the progression of HE is characterized by abnormal functional brain network organization. This is consistent with recent neuroimaging studies^20^,^21^,^39^,^40^,^41^. For example, Jao *et al.*^39^ demonstrated a stepwise deterioration of topological properties in the whole brain network across HE development. Our findings further confirm the continuous nature of HE that is associated with the same pathological mechanism. Therefore, analysis of the triple network mode could potentially be useful for monitoring the progression of HE. Additionally, the correlation between the altered inter-network connectivity and the clinical marker of MHE—PHES further indicates the utility of atypical functional coordination for reflecting the pathologies of MHE and as a complementary biomarker for MHE detection.

This study has several limitations. First, we performed group ICA empirically, because previous studies demonstrated that an ICA model order of approximately 75 components could ensure network stability^15^,^42^, but the selection of the optimal ICA model order to analyze resting-state fMRI data remains an issue of debate. Second, the connectivity patterns among seven sub-networks obtained by high-model-order ICA were inconsistent with several previous reports. The similar phenomenon has been described and explained by Allen *et al.*^31^ and Manoliu *et al.*^15^, but the nature of the interaction among these different intrinsic networks will need to be addressed in the future. Third, the triple network model has demonstrated the role of the SN in initiating network switching between the DMN and CEN, which suggests a controlling effect direction. However, our analysis based on resting-state fMRI cannot provide sufficient evidence regarding the alterations of this functional controlling direction in MHE. A task-related fMRI study may be needed to consider this issue. Finally, the longitudinal studies are recommended to clarify the utility of altered FC as a biomarker for detecting and monitoring HE.

In conclusion, a triple-mode network analysis highlighted the aberrant SN and its functional interaction with the DMN/CEN in MHE, which may underlie the mechanisms of neurocognitive dysfunction in MHE patients. Moreover, the continuous impairment of FC suggests a role of abnormal functional networks in HE progression. Thus, it could be helpful to examine the changes of functional connections among the triple networks for the diagnosis of MHE (individual identification) and monitoring of disease development.

## Additional Information

**How to cite this article**: Chen, H.-J. *et al.* Aberrant salience network and its functional coupling with default and executive networks in minimal hepatic encephalopathy: a resting-state fMRI study. *Sci. Rep.* **6**, 27092; doi: 10.1038/srep27092 (2016).

## Supplementary Material

Supplementary Information

## Figures and Tables

**Table 1:** Demographic and clinical characteristics of the subjects.

	HCs subjects (*n *= 18)	NHE patients (*n *= 23)	MHE patients (*n *= 20)	*P* value (ANOVA)
Age (year)	50.1 ± 6.6	50.8 ± 9.8	49.5 ± 9.2	0.882
Sex (Male/Female)	13/5	19/4	15/5	0.706 (χ^2^-test)
Education level (year)	8.4 ± 2.6	8.2 ± 2.9	8.6 ± 2.8	0.908
Etiology of cirrhosis (HBV/alcoholism/ HBV+alcoholism/other)	–	15/3/2/3	12/3/1/4	
Child–Pugh stage (A/B/C)	-	16/6/1	4/12/4	
PHES test				
Final PHES score	0.44 ± 2.12	−0.87 ± 2.16	−7.80 ± 2.28^*,#^	<0.001
Number connection test A (seconds)	38.2 ± 12.6	39.5 ± 11.7	57.1 ± 14.7^*,#^	<0.001
Number connection test B (seconds)	63.2 ± 31.2	73.3 ± 19.6	120.2 ± 47.8^*,#^	<0.001
Serial dotting test (seconds)	43.3 ± 8.1	46.4 ± 9.1	59.1 ± 8.8^*,#^	<0.001
Digit symbol test (raw score)	44.9 ± 9.3	41.6 ± 11.9	28.9 ± 8.4^*,#^	<0.001
Line tracing test (raw score)	113.8 ± 17.9	148.9 ± 37.0^†^	183.7 ± 37.2^*^	<0.001

Note: MHE, minimal hepatic encephalopathy; NHE, patients without MHE; HCs, healthy controls; PHES, psychometric hepatic encephalopathy score; ANOVA, analysis of variance. The markers *, †, and # respectively indicate the significant difference in neurological performance between MHE and HC, NHE and HC, and MHE and NHE.

**Table 2:** Brain areas with significant differences in the intra-iFC across the three groups.

Regions	Voxels	Brodmann area	MNI coordinates			Peak *F*-value
			x	y	z	
**aDMN**						
Bilateral anterior cingulate cortex	68	24	3	30	15	9.57
**SN**						
Right insular cortex	45	13	39	15	3	9.70
**lvCEN**						
Rihgt inferior parietal lobule	47	39/40	48	−69	39	9.59
**rvCEN**						
Rihgt inferior parietal lobule	67	40/39	48	−63	45	10.73

Notes: aDMN, anterior DMN; SN, salience network; lvCEN, left-ventral CEN; rvCEN, right-ventral CEN.

**Table 3:** Altered inter-network functional connectivity across the three groups.

Inter-iFC	HC subjects	NHE patients	MHE patients	*P* value (ANOVA)
aDMN - ipDMN	0.466 ± 0.197	0.482 ± 0.257	0.364 ± 0.279	0.268
aDMN - spDMN	0.154 ± 0.192	0.168 ± 0.257	0.075 ± 0.268	0.425
aDMN - SN	0.013 ± 0.200	0.279 ± 0.305^†^	0.303 ± 0.330*	**0.005**^‡^
aDMN - lvCEN	0.435 ± 0.228	0.384 ± 0.271	0.334 ± 0.258	0.479
aDMN - rvCEN	0.410 ± 0.166	0.437 ± 0.210	0.440 ± 0.189	0.871
aDMN - dCEN	−0.449 ± 0.214	−0.380 ± 0.210	−0.420 ± 0.262	0.625
ipDMN - spDMN	1.131 ± 0.235	1.133 ± 0.269	1.084 ± 0.311	0.811
ipDMN - SN	−0.494 ± 0.240	−0.231 ± 0.252^†^	−0.168 ± 0.277*	**0.001**^‡^
ipDMN - lvCEN	1.037 ± 0.264	0.949 ± 0.284	1.154 ± 0.220	**0.040**
ipDMN - rvCEN	0.903 ± 0.331	0.846 ± 0.239	0.971 ± 0.277	0.351
ipDMN - dCEN	0.084 ± 0.252	0.072 ± 0.232	0.145 ± 0.203	0.550
spDMN - SN	−0.545 ± 0.223	−0.354 ± 0.269^†^	−0.263 ± 0.215*	**0.002**^‡^
spDMN - lvCEN	0.539 ± 0.271	0.489 ± 0.216	0.617 ± 0.242	0.229
spDMN - rvCEN	0.533 ± 0.293	0.446 ± 0.246	0.503 ± 0.280	0.581
spDMN - dCEN	0.358 ± 0.219	0.318 ± 0.237	0.405 ± 0.168	0.413
SN – lvCEN	−0.320 ± 0.225	−0.092 ± 0.247^†^	−0.056 ± 0.249*	**0.002**^‡^
SN – rvCEN	−0.226 ± 0.279	−0.090 ± 0.269	−0.005 ± 0.271	0.050
SN – dCEN	−0.256 ± 0.237	−0.201 ± 0.251	−0.158 ± 0.244	0.466
lvCEN - rvCEN	0.984 ± 0.304	0.987 ± 0.185	1.034 ± 0.196	0.744
lvCEN – dCEN	0.185 ± 0.252	0.259 ± 0.156	0.261 ± 0.288	0.527
rvCEN – dCEN	0.001 ± 0.213	0.021 ± 0.188	0.071 ± 0.247	0.586

Note: *P* values in bold are <0.05. The marker ^‡^indicates *P* < 0.05 after FDR correction for multiple comparisons. The markers * and ^†^indicate a significant difference in inter-iFC between MHE and HCs and between NHE and HCs, respectively. MHE, minimal hepatic encephalopathy; NHE, patients without MHE; HC, healthy control; ANOVA, analysis of variance. a/ip/spDMN, anterior/inferior-posterior/superior-posterior DMN; lv/rv/dCEN, left-ventral/right-ventral/dorsal CEN; SN, salience network.

**Table 4:** Altered time-lagged inter-network functional connectivity between SN and other networks across the three groups.

Time-lagged inter-network connectivity	HC subjects	NHE patients	MHE patients	*P* value (ANOVA)
**Lag = 1**				
SN → aDMN	0.002 ± 0.177	0.198 ± 0.277^†^	0.223 ± 0.280*	**0.015**^**‡**^
SN → ipDMN	−0.196 ± 0.105	−0.243 ± 0.232	−0.081 ± 0.105*,#	**0.008**^**‡**^
SN → spDMN	−0.220 ± 0.103	−0.357 ± 0.233^†^	−0.118 ± 0.081*,#	**0.001**^**‡**^
SN → lvCEN	−0.113 ± 0.079	−0.073 ± 0.234	−0.029 ± 0.093	0.278
SN → rvCEN	−0.079 ± 0.112	−0.064 ± 0.250	−0.013 ± 0.101	0.468
SN → dCEN	−0.092 ± 0.097	−0.167 ± 0.250	−0.065 ± 0.094	0.138
**Lag = 2**				
SN → aDMN	0.026 ± 0.151	0.108 ± 0.236	0.112 ± 0.212	0.070
SN → ipDMN	−0.325 ± 0.192	−0.198 ± 0.192^†^	−0.153 ± 0.195*	**0.015**^**‡**^
SN → spDMN	−0.377 ± 0.186	−0.301 ± 0.189	−0.211 ± 0.148*	**0.016**^**‡**^
SN → lvCEN	−0.169 ± 0.132	−0.029 ± 0.207	−0.054 ± 0.176	**0.041**
SN → rvCEN	−0.113 ± 0.215	−0.019 ± 0.215	−0.035 ± 0.181	0.322
SN → dCEN	−0.151 ± 0.209	−0.119 ± 0.234	−0.113 ± 0.188	0.833
**Lag = 3**				
SN → aDMN	−0.052 ± 0.140	0.020 ± 0.198	0.008 ± 0.160	0.376
SN → ipDMN	−0.160 ± 0.148	−0.120 ± 0.154	−0.097 ± 0.170	0.476
SN → spDMN	−0.193 ± 0.135	−0.200 ± 0.143	−0.117 ± 0.137	0.115
SN → lvCEN	−0.067 ± 0.120	0.018 ± 0.173	−0.036 ± 0.158	0.206
SN → rvCEN	−0.042 ± 0.173	0.020 ± 0.175	−0.039 ± 0.152	0.392
SN → dCEN	−0.058 ± 0.193	−0.057 ± 0.206	−0.058 ± 0.166	0.980

Note: *P* values in bold are <0.05. The marker ^‡^indicate *P* < 0.05 after FDR correction for multiple comparisons. The markers *, †, and ^#^indicate significant differences between MHE and HCs, NHE and HCs, and NHE and MHE, respectively. MHE, minimal hepatic encephalopathy; NHE, patients without MHE; HC, healthy control; ANOVA, analysis of variance. a/ip/spDMN, anterior/inferior-posterior/superior-posterior DMN; lv/rv/dCEN, left-ventral/right-ventral/dorsal CEN; SN, salience network.
