# Supplementary material for: Aberrant salience network and its functional coupling with default and executive networks in minimal hepatic encephalopathy: a resting-state fMRI study
Source: Sci Rep. 2016 Jun 1;6:27092. doi: 10.1038/srep27092 (PMC4890427; doi:10.1038/srep27092)

Aberrant salience network and its functional coupling with default and executive networks in minimal hepatic encephalopathy: a resting-state fMRI study

Hua-Jun Chen[1,*], Qiu-Feng Chen[2], Jun Liu[1], Hai-Bin Shi[1,*]

[1] Department of Radiology, The First Affiliated Hospital of Nanjing Medical University, Nanjing 210029, China

[2] School of Information Science and Engineering, Central South University, Changsha 410083, China

***Corresponding author:**

Hua-Jun Chen, MD; Hai-Bin Shi, MD

Department of Radiology, The First Affiliated Hospital of Nanjing Medical University, Nanjing 210029, China

Tel.: +86 25 68137519; fax: +86 25 68137518

E-mail addresses: chj0075@126.com; shihb@vip.sina.com

Time-lagged inter-iFC

SN is an integral hub in mediating dynamic activities between the CEN and DMN. We hypothesized an altered temporal interaction between SN's preceding TC and DMN/CEN TCs in MHE. A time-lagged correlation analysis was performed to investigate temporal dependencies between the SN and CEN/DMN subsystems. A pairwise correlation analysis was applied on SN´s centered TC and TCs of DMN/CEN, with a time shift of 1 to 3 time points (i.e., $SN_{t(i0)} \rightarrow DMN/CEN_{t(i0+ \Delta i)}$ for i0 = 1 to n time points and Δi = 1-3 time points). The Pearson's correlation coefficients obtained were then transformed to Z-scores using Fisher's Z-transformation and compared across the three groups, with age, gender, education level, and GM volumes as covariates of no interest ($P < 0.05$, FDR correction).

Supplementary Figure legend

Supplementary Figure 1. Differences in the mean intra-iFC of the entire intrinsic network. The intra-iFC was measured using the Z value retrieved by independent component analysis. The marker ‡ indicates the intrinsic network in which the intra-iFC is significantly different across the three groups ($P < 0.05$, by one-way analysis of variance). The markers *, †, and # indicate significant differences of intra-iFC between MHE and HCs, NHE and HCs, and NHE and MHE, respectively. MHE, minimal hepatic encephalopathy; NHE, patients without MHE; HCs, healthy controls. a/ip/spDMN, anterior/inferior-posterior/superior-posterior DMN; lv/rv/dCEN, left ventral/right ventral/dorsal CEN; SN, salience network. It is noted that MHE patients showed decreased trends in intra-iFC for all DMN, SN, and CEN networks.

Supplementary Figure 1

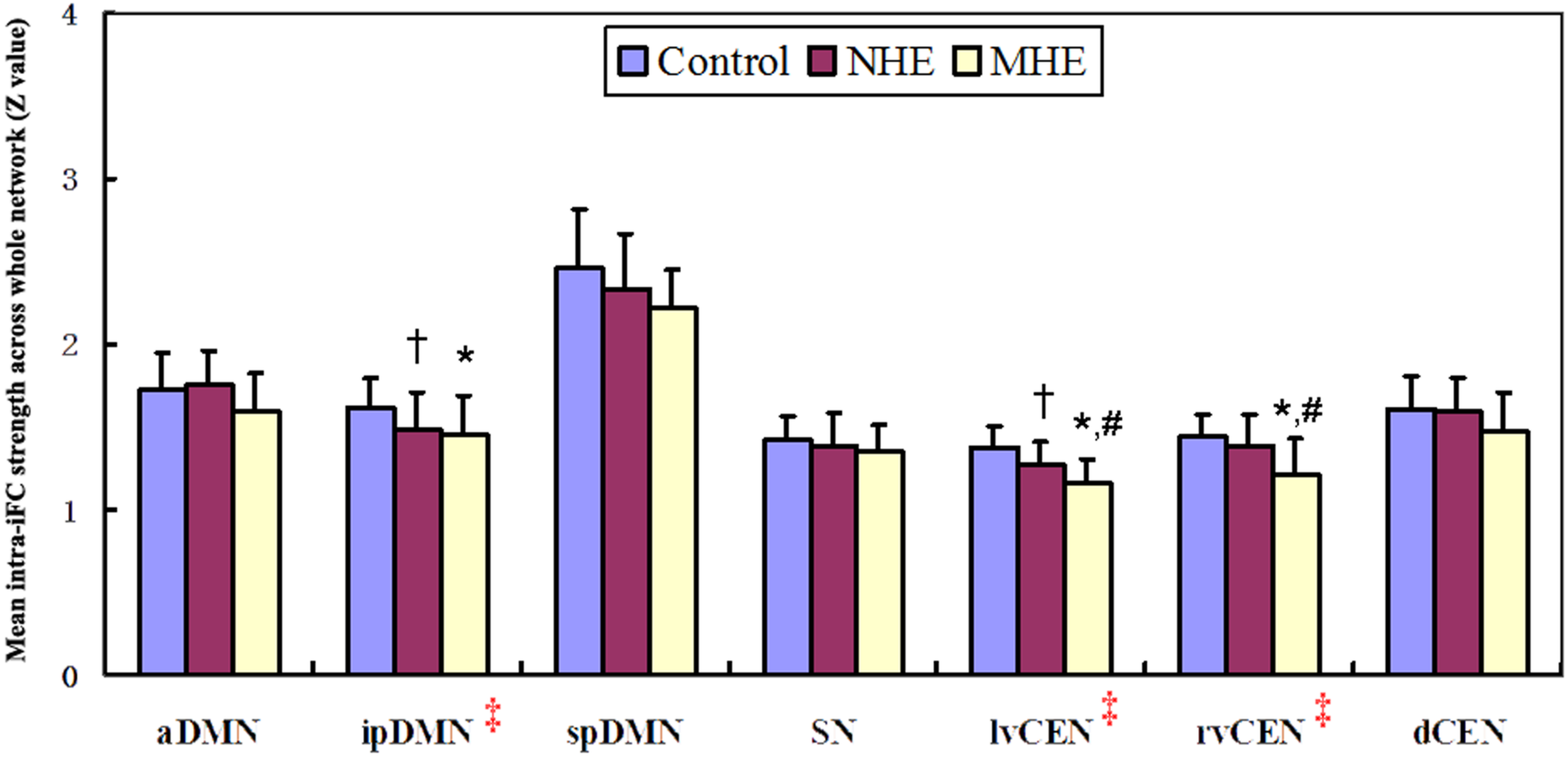

Supplement: Supplementary Information [file srep27092-s1.pdf]
